# Development of a Triple-Negative Breast Cancer Leptomeningeal Disease Model in Zebrafish

**DOI:** 10.3390/cells12070995

**Published:** 2023-03-24

**Authors:** Udhayakumar Gopal, Jerry D. Monroe, Amarnath S. Marudamuthu, Salma Begum, Bradley J. Walters, Rodney A. Stewart, Chad W. Washington, Yann Gibert, Marcus A. Zachariah

**Affiliations:** 1 Department of Neurosurgery, University of Mississippi Medical Center, Jackson, MS 39216, USA; 2 Department of Cell and Molecular Biology, Cancer Center and Research Institute, University of Mississippi Medical Center, Jackson, MS 39216, USA; 3 Department of Otolaryngology-Head and Neck Surgery, University of Mississippi Medical Center, Jackson, MS 39216, USA; 4 Department of Oncological Sciences, Huntsman Cancer Institute, University of Utah, Salt Lake City, UT 84112, USA; 5 Neurosurgical Medical Clinic, 3750 Convoy Street, Suite 301, San Diego, CA 92111, USA

**Keywords:** triple-negative breast cancer, zebrafish, leptomeningeal disease, xenograft, doxorubicin

## Abstract

Leptomeningeal disease occurs when cancer cells migrate into the ventricles of the brain and spinal cord and then colonize the meninges of the central nervous system. The triple-negative subtype of breast cancer often progresses toward leptomeningeal disease and has a poor prognosis because of limited treatment options. This is due, in part, to a lack of animal models with which to study leptomeningeal disease. Here, we developed a translucent zebrafish *casper* (*roy*-/-; *nacre*-/-) xenograft model of leptomeningeal disease in which fluorescent labeled MDA-MB-231 human triple-negative breast cancer cells are microinjected into the ventricles of zebrafish embryos and then tracked and measured using fluorescent microscopy and multimodal plate reader technology. We then used these techniques to measure tumor area, cell proliferation, and cell death in samples treated with the breast cancer drug doxorubicin and a vehicle control. We monitored MDA-MB-231 cell localization and tumor area, and showed that samples treated with doxorubicin exhibited decreased tumor area and proliferation and increased apoptosis compared to control samples.

## 1. Introduction

Leptomeningeal disease (LMD) occurs when cancer cells metastasize from an original tumor site into the cerebrospinal fluid (CSF) that occupies the subarachnoid space between the pia and arachnoid mater layers of the meninges that surround the central nervous system [1,2,3,4]. LMD comprises up to 8% of solid and 15% of hematological malignancies, with over 100,000 new cases annually in the United States, and has a very poor prognosis with a median survival of 3–16 weeks despite treatment [5,6]. Breast cancer is the most common solid tumor associated with LMD, and its triple-negative subtype (TNLMD) demonstrates the highest propensity to metastasize to the leptomeninges [7]. With few exceptions, e.g., [8], clinical trials of new drug candidates exclude patients with LMD, placing a major barrier to improvement in patient care. Additionally, investigation of LMD in mouse models has been hindered by high cost, low throughput, and the need for millions of cells for injection, as well the models’ inherent opacity, which can restrict the ability to visually analyze cancer proliferation, metastasis, and response to chemotherapeutics using fluorescent labeling techniques [9,10,11]. As a result, the mechanisms that modulate LMD-associated cancer cell growth, translocation, and drug response through the CSF and associated structures remain incompletely understood.

The optical clarity of the zebrafish (*Danio rerio*) model makes it uniquely suited for the study of TNLMD as it enables microinjection of cancer cells into LMD-associated anatomical structures while allowing simultaneous fluorescent monitoring of cell proliferation and migration [12,13]. A major advantage of zebrafish xenograft models over rodent models is the availability of pigment-deficient strains, e.g., *casper* (*roy*-/-; *nacre*-/-), that allow observation of transplanted cancer cells, and the absence until 4–6 weeks after fertilization of an adaptive immune system that could reject foreign implanted cells [11,14,15]. The primary conduit for the spread of LMD is the CSF found in the interconnected subarachnoid space and the ventricular system associated with the brain and spinal cord [1,2]. During zebrafish development, the ventricular system first forms between 17 and 24 h postfertilization (hpf) and further expands from 24 to 36 hpf after the onset of the heartbeat and circulation [16,17]. Microinjection of fluorescent rhodamine dye into the CSF of the hindbrain ventricle permits microscopic visualization and analysis of the developing zebrafish ventricular system [16,17,18]. Researchers have microinjected glioblastoma cells labeled with the red fluorescent dye 1,1′-dioctadecyl-3,3,3′,3′-tetramethylindocarbocyanine (DiI) into the zebrafish hindbrain ventricle and assessed cancer cell proliferation and migration using bright-field and fluorescent microscopy, and analyzed glioblastoma response to the phosphoinositide 3-kinase inhibitor LY294002, zinc oxide nanoparticles, and isothiocyanate derivatives [12,13]. In addition, transplantation of zebrafish brain tumor cells into the fourth ventricle resulted in LMD along the spinal cord in a subset of recipients [19]. These studies suggest that the zebrafish xenograft model is potentially well suited for the investigation of LMD mechanisms and response to chemotherapeutics.

Here, we adapted a zebrafish embryo intraventricular microinjection protocol [12,15] to establish the first zebrafish xenograft embryo model of TNLMD. This model uses the triple-negative breast cancer cell line MDA-MB-231/Luc-RFP, a transgenic reporter breast cancer cell line that allows the detection of cells via red fluorescence expression, which has been used to study cancer xenograft proliferation and migration in zebrafish models [20]. Also tested were DiI-labeled MDA-MB-231 and MCF-10A cell lines, which have been successfully used in comparative studies assessing the cell proliferation and migration of normal breast tissue (MCF-10A) and TNBC (MDA-MB-231) cells in zebrafish xenograft models [21]. We first microinjected dyes or fluorescent labeled TNBC cells into the fourth ventricles of zebrafish embryos and used microscopy and cross-section staining to assess whether microinjected materials would initially localize to the cranial ventricular space. Then, we devised a protocol to measure the area of fluorescent TNBC xenografts in the zebrafish by calculating their corrected total cell fluorescence (CTCF) values at selected imaging time points, and also used a multimodal plate reader to monitor TNBC cell growth in the xenograft samples. Next, we used the model to quantify and compare MDA-MB-231 breast cancer and MCF-10 normal breast tissue proliferation. TNBC xenograft samples were treated with either doxorubicin, a drug used in TNBC breast cancer treatment [22], or dimethyl sulfoxide (DMSO) vehicle, and the effect of treatment on tumor area was analyzed. We also assessed the effects of the treatments on proliferation via Ki67 staining and apoptosis with anti-cleaved-caspase-3 antibody staining using z-stack analysis [23,24]. We found that we were able to monitor and measure TNBC cell proliferation in the ventricular space associated with TNLMD over several time points and track secondary tumor migration. Further, doxorubicin treatment decreased TNBC cancer area and proliferation, while it promoted TNBC cell apoptosis in the xenograft samples. These results show that the zebrafish xenograft TNLMD model is a valuable new tool that considerably advances multimodal visual analysis of TNBC tumor behavior and pharmaceutical treatment response during leptomeningeal disease.

## 2. Materials and Methods

### 2.1. Zebrafish Maintenance

Transparent *casper* (*roy*-/-; *nacre*-/-) zebrafish were obtained from the Zebrafish International Resource Center (Cat. #: ZL1714). Zebrafish were maintained at 28 °C with a 14 h light and 10 h dark cycle according to established protocols [25]. All zebrafish experiments were conducted in accordance with the guidelines of the Animal Care and Use Committee of the University of Mississippi Medical Center (UMMC) Jackson, MS, USA, and approved by the UMMC Institutional Biosafety and IACUC Review Committees (IACUC protocol number: 2021-1161).

### 2.2. Cell Culture

The triple-negative breast cancer (TNBC) cell line MDA-MB-231/Luc-RFP (a kind gift from the Dr. Gene L. Bidwell lab, UMMC), MDA-MB-231 cell line, and MCF10A cell line were obtained from the American Type Culture Collection (Manassas, VA, USA). MDA-MB-231/Luc-RFP and MDA-MB-231 cells were cultured in Dulbecco’s modified Eagle’s medium (DMEM) (Gibco, Gaithersburg, MD, USA) containing 10% fetal bovine serum (FBS) (Gibco) with 1:100 penicillin/streptomycin (Invitrogen, Carlsbad, CA, USA) and incubated at 37 °C with 5% CO_2_. MDA-MB-231/Luc-RFP cells were selected using blasticidin (Gibco). MCF10A cells were cultured in DMEM/F12 medium (Gibco) with supplementation as for the TNBC cell lines.

### 2.3. Zebrafish Xenograft Microinjection

Prior to microinjection, approximately 150,000 TNBC cells were collected and suspended in phosphate-buffered saline (PBS) (Gibco) and then treated with DiI (Invitrogen) according to the manufacturer’s protocol, loaded into a borosilicate glass needle (World Precision Instruments, Sarasota, FL, USA) prepared using a Narishige PN-30 micropipette puller (Amityville, NY, USA), and then placed into a Narishige stereotaxic apparatus for injection. Zebrafish larvae at 2 days postfertilization (dpf) were anesthetized with 0.2 mg/mL tricaine methanesulfonate (MS-222) (Sigma-Aldrich, St. Louis, MO, USA) and placed into a glass injection tray with E3 water (5 mM NaCl, 0.17 mM KCl, 0.33 mM CaCl_2_, 0.33 mM MgSO_4_, 1 ppm methylene blue) (Sigma), and approximately 100 cells were then injected into the fourth ventricles of individual larvae using a Tritech Research microinjector (Los Angeles, CA, USA) and Labomed Luxeo 6z stereo microscope (Fremont, CA, USA). Injected larvae were then placed into a 28 °C incubator, a temperature shown in trial experiments to not negatively influence cancer cell proliferation or viability, for 24 h. Larvae were then microscopically examined and those exhibiting cellular debris or fewer than 100 labeled cells in the fourth ventricle were discarded, while larvae with at least 100 tumor cells in the fourth ventricle were retained for future study. To assess tumor cell localization in xenograft samples, a set of six vehicle-microinjected and another set of six TNBC-microinjected larvae were prepared and then, at 8 dpi, all larvae were placed in Dietrich’s fixative (Fisher Scientific, Hampton, NH, USA), embedded in paraffin, and sectioned at a thickness of 3 µm. Sections were then stained with hematoxylin and eosin (Fisher Scientific; Cat. #: 72704) for histopathological analysis. To image ventricles, a set of six sample larvae were injected with dextran fluorescein (Invitrogen) and DiI-treated TNBC cells and fluorescent imaged using a Zeiss LSM 880 confocal microscope (Jena, Germany), with image acquisition achieved using Zen black 2.3 S1 and image analysis achieved using Zen blue 2.6.

### 2.4. Drug Treatment and Tumor Size Assay

Different concentrations of doxorubicin (Selleck Chemicals, Houston, TX; Cat. #: S1208) were prepared in dimethyl sulfoxide (DMSO) (Thermo Fisher, Waltham, MA, USA) solvent and then tested *in vivo* and *in vitro* to determine the zebrafish maximum tolerable concentration and effective anticancer cell culture concentrations. We found that doxorubicin at 8 µM exhibited an anticancer effect in the cell lines and was well-tolerated by zebrafish larvae, and we used this value for subsequent experiments. Further, this value, 8 µM, is very similar to the reported peak clinical human plasma concentrations, e.g., 4 µg/mL (7 µM) [26]. Next, zebrafish xenografts with the same tumor sizes were randomly distributed into treatment and control groups after 1 dpi. Doxorubicin or DMSO control was added to the fish water, and the water was changed daily with fresh drug applied each day until 8 dpi. Tumor area in zebrafish xenografts was measured using a BioTek Cytation 7 multimodal imager and plate reader (Winooski, VT, USA) at 2, 4, and 8 dpi. As autofluorescence was not detected in the doxorubicin controls, tumor size was then determined by quantifying the two-dimensional image area using ImageJ (National Institutes of Health, Bethesda, MD, USA) and the following formula: corrected total cell fluorescence (CTCF) = integrated density − (area of selected fluorescent region of interest × background mean gray value). For experiments in which tumor migration was tracked, we used transparent AB wild-type zebrafish embryos, a reference strain which readily permits fluorescent monitoring of tumor metastasis, instead of *casper* fish.

### 2.5. Immunofluorescence Assay

Treated and control xenograft larvae were euthanized with 25X MS-222 and then fixed with PIPES (Sigma) solution for 24 h, which was subsequently replaced with 100% methanol followed by storage at −20 °C. Samples were then rehydrated using a series of decreasing methanol concentrations (75%, 50%, 25%) diluted in PBS/0.1% Triton X-100 (Thermo Fisher, Cat. #: A16046.AE). Larvae were then washed four times for 5 min in PBS/0.1% Triton X-100, followed by washing once for 5 min in water which was then removed and replaced with ice-cold acetone before incubation at −20 °C for 7 min. Samples were then washed again twice for 10 min in PBS/0.1% Triton X-100 and incubated with PBDX_GS blocking solution (for 50 mL, PBS 1X: 0.5 mL of DMSO, 250 μL of 10% Triton X-100, 0.5 g of bovine serum albumin (Thermo Fisher), and 750 μL of 15 μL/mL goat serum (Sigma)) [27] for 1 h at room temperature (RT). Primary Ki-67 antibody conjugated with Alexa Fluor 488 (Cell Signaling Technology, Danvers, MA, USA; Cat. #: 11882) diluted (1:10) in PBDX_GS was added and incubated for 1 h at room temperature and then at 4 °C overnight. Samples were next washed four times for 15 min with PBS/0.05% Tween which was then removed and replaced with 4′,6-diamidino-2-phenylindole (DAPI) (Invitrogen) (1:10,000) followed by incubation for 15 min at RT and washing four times for 5 min in PBS/0.05% Tween. The PBS/0.05% Tween solution was removed and 1 drop of aqueous 0.02% methyl cellulose (Sigma) mounting medium was added to each microcentrifuge tube, with samples then stored at 4 °C for future imaging. Fluorescent and bright-field images were taken with a Zeiss LSM 880 using the Airyscan z-stack function at 20× magnification with a 5 µm interval between each slice. For caspase-3 staining, the same protocol was used as for the Ki-67 staining except that instead of Ki-67 antibody, primary cleaved caspase-3 antibody conjugated with Alexa Fluor 488 (Cell Signaling Technology; Cat. #: 9669) and diluted 1:25 was used.

### 2.6. Statistical Analysis

In all experiments, zebrafish were randomly assigned to experimental groups. GraphPad PRISM version 9 software (La Jolla, CA, USA) was used for all data representation and statistical analysis. Datasets were either analyzed with a two-way ANOVA followed by either Dunnett’s or Tukey’s multiple-comparisons test, or an unpaired two-tailed Student’s *t*-test. A *p* value of ≤0.05 was considered statistically significant for all experiments.

## 3. Results

**Figure 1:**
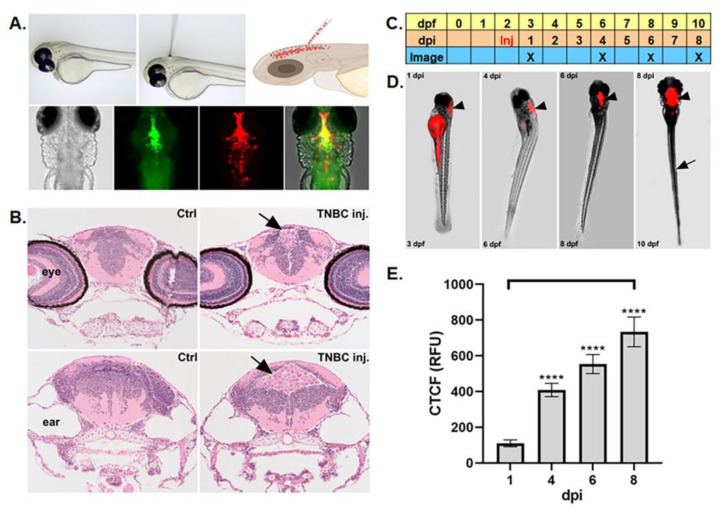
Microinjection of fluorescent labeled TNBC cells into the zebrafish hindbrain ventricle facilitated measurement of tumor area and metastasis. (**A**). Top row left: lateral view of zebrafish embryo prior to injection; top row center: lateral view of embryo injection; top row right: diagram of zebrafish microinjection with cancer cells indicated in red; bottom row far left: dorsal bright-field view of zebrafish cranial region; bottom row inner left: dorsal view after microinjection with green dextran fluorescein dye (to label ventricle); bottom row inner right: dorsal view after microinjection with red DiI-labeled TNBC cells; bottom row far right: dorsal view of red–green color combination. (**B**). Hematoxylin and eosin staining cross-sectional study showing rostral view (top row) and caudal view (bottom row) comparing control uninjected samples (left panels) with ventricle MDA-MB-231 TNBC-injected samples (right panels; injected TNBC cells indicated in different brain ventricles with arrows) (**C**). Schematic timeline for experiments showing days postfertilization (dpf), days postinjection (dpi), injection time (Inj), and imaging time points. (**D**). Fluorescent tracking of MDA-MB-231-Luc/RFP labeled TNBC cells over 1, 4, 6, and 8 dpi (arrow heads: labeled cancer cells localized in hindbrain; arrow: labeled cancer cells in caudal section). (**E**). Measurement of corrected total cell fluorescence at 1, 4, 6, and 8 dpi. Key: corrected total cell fluorescence (CTCF); *p* < 0.05, “****” = 0.0001; Error bars = SEM; N for 1, 4, and 6 dpi samples = 44; N for 8 dpi samples = 29.

**Figure 2:**
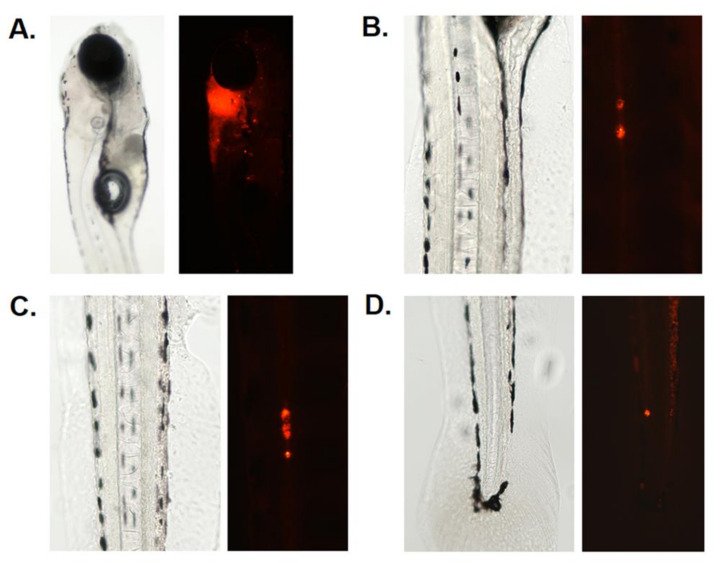
Fluorescent tracking of secondary tumor migration in zebrafish MDA-MB-231 TNBC xenografts. (**A**). Bright-field view of a 6 dpf AB zebrafish larvae (left) and fluorescent channel image of larvae (right) microinjected with DiI-treated MDA-MB-231 TNBC cells at 4 dpi. (**B**–**D**). Bright-field view (left) and fluorescent view (right) of caudal sections of migrated MDA-MB-231 TNBC cells in the spinal cord of AB zebrafish larvae at 8 dpi. (**B**): anterior; (**C**): medial; (**D**): posterior caudal section of the zebrafish larvae.

The transparent *casper* (*roy*-/-; *nacre*-/-) zebrafish line was evaluated to determine whether it possessed suitable properties to allow identification of, access to, and imaging of the ventricles of the central nervous system (CNS) using fluorescent dyes and microscopy techniques [18,28] (Figure 1A). MDA-MB-231 triple-negative breast cancer cells were microinjected into the zebrafish fourth ventricle and cross-sectional analysis was performed on control (noninjected) and TNBC-injected samples using hematoxylin and eosin staining [29,30]. This analysis showed that TNBC-injected samples had ventricles populated with cancer cells (Figure 1B arrows; Video S1). We then developed a time course schema for microinjection of reporter-expressing or dye-treated cancer cells to optimize the following parameters: days postfertilization (dpf) of the host, days postinjection (dpi), injection time (Inj), and four discrete imaging time points (Figure 1C). Following this temporal schema, MDA-MB-231 TNBC cells treated with DiI were microinjected into the fourth ventricle and the area occupied by cancer cells in the ventricular space was monitored using epifluorescence microscopy over four time points (1, 4, 6, 8 dpi), which showed that labeled cancer cell area increased in the ventricular space (arrow heads, Figure 1D). We also identified metastasized TNBC cells at locations distant from the injection site (arrow, Figure 1D; see Figure 2). Tumor cell area in the ventricle was measured using Image J (National Institutes of Health, Bethesda, MD, USA). To eliminate the effect of autofluorescence, which can be expressed in yolk sac lipids [31] (see Figure 1D, lateral view of yolk sac at 1 dpi), we calculated corresponding corrected total cell fluorescence (CTCF) values, a technique used to subtract background fluorescence in zebrafish cancer models [32], and found that the area occupied by TNBC cells increased over the 1, 4, 6, and 8 dpi time points (mean CTCF values in relative fluorescence units (RFU): 110.25 (1 dpi), 408.99 (4 dpi), 553.55 (6 dpi), 734.01 (8 dpi); Figure 1E). To further analyze whether the model would allow for monitoring of fluorescent secondary tumor cell migration in the CSF-containing compartments associated with the zebrafish spinal cord, we also microinjected DiI-labeled MDA-MB-231 TNBC cells into the fourth ventricles of AB wild-type zebrafish and then photographically identified the tumors that formed in the anterior, medial, and posterior caudal sections of the larvae at 8 dpi (Figure 2B–D).

**Figure 3:**
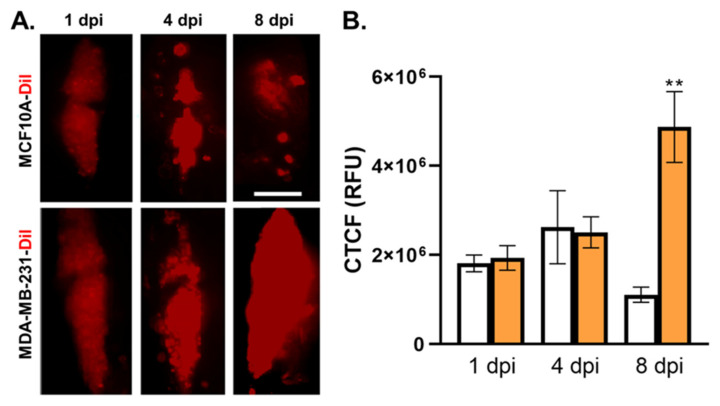
MDA-MB-231 TNBC cells exhibited increased tumor area in the zebrafish hindbrain ventricle compared to noncancerous breast tissue cells. (**A**). Top row: images of noncancerous breast tissue MCF10A xenograft cells treated with DiI in the hindbrain ventricle at 1, 4, and 8 dpi; bottom row: images of cancerous breast tissue MDA-MB-231 xenograft cells treated with DiI in the hindbrain ventricle at 1, 4, and 8 dpi. (**B**). Comparison of MCF10A (white columns) and MDA-MB-231 (orange columns) corrected total cell fluorescence values at 1, 4, and 8 dpi. Key: days postinjection (dpi); corrected total cell fluorescence (CTCF); relative fluorescence units (RFU); *p* < 0.05, “**” = 0.01; Error bars = SEM; scale bar = 100 µm; N for MCF10A: 27 (1 dpi), 30 (4 dpi), 33 (8 dpi); N for MDA-MB-231: 27 (1 and 4 dpi), 35 (8 dpi).

To assess whether cancerous and noncancerous breast tissue cells exhibited distinct growth characteristics in the ventricular space, we microinjected zebrafish ventricles with either DiI-labeled MDA-MB-231 TNBC cells or DiI-labeled noncancerous MCF-10A breast-tissue-derived cells. We imaged the areas of the brain using fluorescent staining at 1, 4, and 8 dpi (Figure 3A) to measure the CTCF values for the two cell types. We found that the TNBC cells exhibited increased CTCF values: 1,931,386.931 (1 dpi), 2,509,178.89 (4 dpi), and 4,872,644.312 (8 dpi); in contrast, the values for the MCF-10A cells were 1,811,513.037 (1 dpi), 2,621,735.8 (4 dpi), and 1,107,369.469 (8 dpi). These measurements show that the TNBC cells, but not noncancerous cells, exhibited expansion in the hindbrain by 8 dpi, which indicated proliferative growth (Figure 3B).

**Figure 4:**
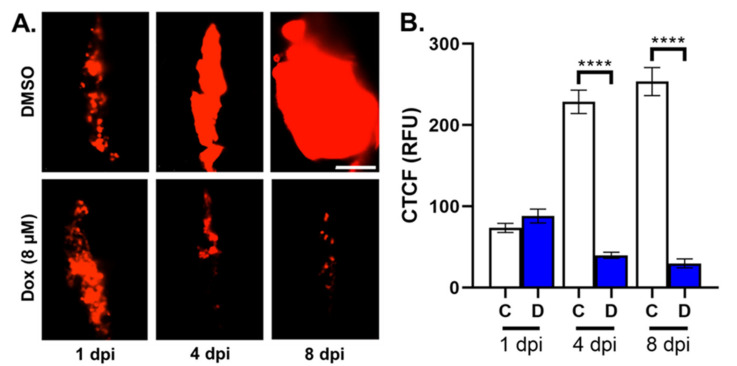
Doxorubicin treatment decreased tumor area compared to DMSO vehicle in zebrafish MDA-MB-231 TNBC xenografts. (**A**). Top row: images showing the area occupied by MDA-MB-231-Luc/RFP TNBC cells in zebrafish xenograft samples treated with DMSO vehicle at 1, 4, and 8 days postinjection; bottom row: images showing the area occupied by MDA-MB-231-Luc/RFP TNBC cells in zebrafish xenograft samples treated with 8 µM doxorubicin at 1, 4, and 8 dpf. (**B**). Measurement of corrected total cell fluorescence for DMSO-treated samples (white columns) and doxorubicin-treated samples (blue columns) at 1, 4, and 8 dpi. Key: CTCF = corrected total cell fluorescence; RFU = relative fluorescence units; Dox = doxorubicin. N = 3 biological replicates with 26–30 total technical replicates. “****” = *p* < 0.0001; Error bars = SEM. Scale bar = 200 µm.

**Figure 5:**
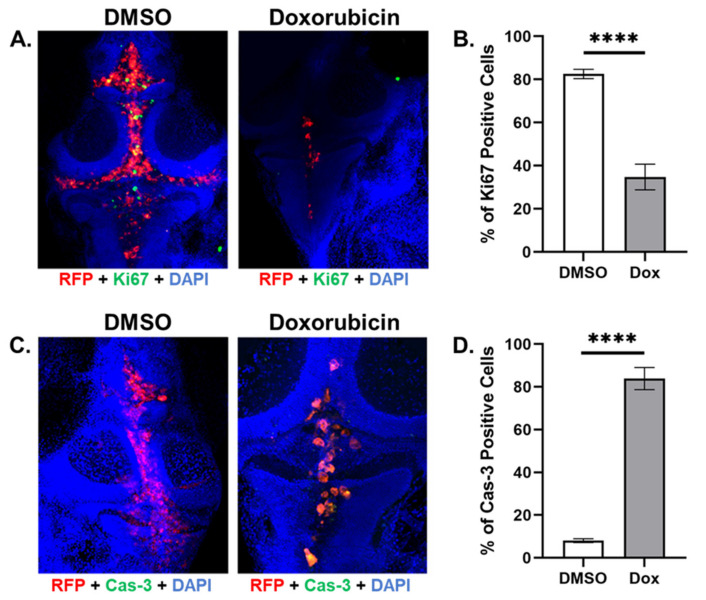
Doxorubicin treatment decreased cell proliferation and increased cell death compared to DMSO vehicle in zebrafish MDA-MB-231 TNBC xenografts. (**A**). Z-stack analysis combining red (MDA-MB-231-Luc/RFP cells), green (Ki67-stained cells), and blue (DAPI-nuclear-stained cells) filter images show that xenograft samples treated with DMSO vehicle exhibited more Ki67 staining than samples treated with doxorubicin at 8 dpi. (**B**). Doxorubicin treatment reduced the percentage of Ki67-positive cells compared to DMSO-vehicle-treated cells at 8 dpi. (**C**). Z-stack analysis combining red (MDA-MB-231-Luc/RFP cells), green (cleaved-caspase-3-stained cells), and blue (DAPI-nuclear-stained cells) filter images show that xenograft samples treated with DMSO vehicle exhibited more cleaved caspase-3 staining than samples treated with doxorubicin at 8 dpi. (**D**). Doxorubicin treatment increased the percentage of cleaved-caspase-3-positive cells compared to DMSO-vehicle-treated cells at 8 dpi. Key: RFP = red fluorescent protein; DAPI = 4′,6-diamidino-2-phenylindole; Cas-3 = caspase-3; Dox = doxorubicin. N = 10; “****” = *p* < 0.0001; Error bars = SEM.

In order to test whether the zebrafish model could be used to assess the effect of anticancer drugs on TNBC cells during LMD, we microinjected zebrafish ventricles with DiI-labeled MDA-MB-231 TNBC cells and then imaged the fluorescent tumor area in samples treated either with the anticancer drug doxorubicin, or DMSO vehicle at 1, 4, and 8 dpi (Figure 4A). CTCF values were measured for the two treatment categories and samples treated with 8 µM doxorubicin exhibited decreased tumor area compared to controls at 4 and 8 dpi (CTCF in RFU at 4 dpi (DMSO: 228.80, doxorubicin: 39.88), 8 dpi (DMSO: 253.63, doxorubicin: 29.90); Figure 4B). Z-stack analysis was then performed in conjunction with fluorescent imaging of RFP-expressing MDA-MB-231 cells (red channel), fluorescent staining of the cell proliferation marker Ki67 (green channel), and the fluorescent nuclear stain DAPI (blue channel), in order to measure actively proliferating TNBC cells (Figure 5A) and the effects of DMSO and doxorubicin on MDA-MB-231 cell proliferation. We found that doxorubicin treatment caused cell proliferation to decrease compared to DMSO vehicle (percent of Ki67-positive cells in cells expressing both DAPI and RFP: DMSO, 82.56%; doxorubicin, 34.74%; Figure 5B). We then repeated z-stack analysis using RFP (red channel) and DAPI (blue channel) with cleaved caspase-3 staining (green channel) to identify apoptotic TNBC cells (Figure 5C) and measure the effects of DMSO and doxorubicin on MDA-MB-231 cell death, and found that doxorubicin increased the percentage of apoptotic cells compared to DMSO vehicle (percent of caspase-3-positive cells in cells expressing both DAPI and RFP: DMSO, 8.03%; doxorubicin, 83.90%; Figure 5D).

## 4. Discussion

### 4.1. The Zebrafish Xenograft Model Facilitates the Study of TNLMD

In this project, we sought to develop a zebrafish model of TNLMD that successfully combines the biological characteristics of leptomeningeal disease with analytical accessibility. Studies of LMD in human patients show that breast cancer cells can metastasize through arterial or venous vessels and along peripheral nerves, perivascular spaces, direct expansions of parenchymal cerebral metastases, and the lymphatic system, to the CSF, which allows further cancer dissemination throughout the structures of the central nervous system including the brain and spinal cord [33,34,35]. Neuroimaging studies of cancer patients have shown that the ventricles are an important site of LMD cancer metastatic implantation and act as a conduit for further spread through the CSF [36]. Analysis of samples taken from CSF is the primary means of detecting LMD [37], with enlargement of the fourth ventricle being diagnostically associated with LMD [38]. The fourth ventricle shares CSF with the cisterna magna [39,40], and mouse models of LMD have shown that seeding of the leptomeninges with fluorescent magnetic-nanoparticle-labeled medulloblastoma (MB) cells can be performed by injecting the cells into the cisterna magna with subsequent monitoring of cell spreading behavior to the leptomeninges accomplished using bioluminescent imaging technology [41]. However, histopathological analysis is still required to assess cancer cell colonization of the leptomeninges and perform molecular studies [41]. Histopathological analysis is time-consuming and may miss dynamic attributes of LMD dissemination and colonization, because of an inability to directly observe cell behaviors *in vivo*.

The zebrafish hindbrain fourth ventricle becomes apparent at the 25-somite stage (21.5 hpf), and the fluid-filled central canal of the zebrafish spinal cord begins to appear at the 21-somite stage (19.5 hpf) [42]. MDA-MB-231 TNBC cells labeled with GFP have been injected into the vasculature of transparent transgenic *Tg* (*mpeg1*:*mCherry*) zebrafish embryos and then studied for extravasation, metastasis, and immune system interaction [43], but to our knowledge, injection of TNBC cells into ventricle structures associated with LMD and their study has not been attempted. The data that we obtained via dye imaging, cross-section staining analysis, and measurement of TNBC tumor area and spinal cord metastasis, suggest that the zebrafish hindbrain ventricle compartment is an accessible and tractable structure for the analysis of TNBC cell proliferation and metastasis (Figure 1 and Figure 2; Video S1).

The leptomeningeal microenvironment is poor in nutrients and growth factors and is typically hypoxic [44] relative to other sites of metastasis. Our data showed that only the MDA-MB-231 TNBC cells, but not the noncancerous breast-tissue-derived MCF10A cells, were able to proliferate in the hindbrain ventricle (Figure 3), suggesting that TNBC cells may be able to sustain cell proliferation and/or outcompete other cells for nutrients in the ventricles, as has been reported to occur between brain tumor tissue and immune cells in hypoxic environments [45].

### 4.2. The Zebrafish Model Can Successfully Validate Drug Efficacy against TNLMD

The TNLMD zebrafish xenograft model can also facilitate fluorescent microscopic analysis of marker proteins involved in TNBC proliferation and cell death in response to drug treatment. Doxorubicin is an anticancer drug used to treat neural cancers, and exosome-encapsulated forms of this drug have been developed that can cross the zebrafish blood–brain barrier in *Tg* (*fli1*:*EGFP*) zebrafish and reduce xenografted U87 glioblastoma tumor volume [46]. Green-fluorescent-protein-labeled MDA-MB-231 TNBC cells have also been microinjected into zebrafish larvae followed by treatment with liposome-encapsulated doxorubicin, which caused decreased tumor cell mass [47]. As we found that doxorubicin treatment decreased tumor area compared to vehicle treatment (Figure 4), these data suggest that the TNLMD model in conjunction with fluorescent plate reading techniques can be used to evaluate how drugs affect LMD progression. Analysis of tumor proliferation using Ki67 staining in zebrafish MDA-MB-231 xenograft models has also been used in studies of cancer cell migration [48], and fluorescence sensing of caspase-3 expression has been used to analyze the role of natural killer cells in promoting apoptosis in *Tg* (*fli1*:*EGFP*) zebrafish embryos [49]. As we found that we could use Ki67 and cleaved caspase-3 staining to show that doxorubicin treatment caused decreased cell proliferation and increased cell death compared to vehicle treatment (Figure 5; Videos S2–S5), these data suggest that the zebrafish TNLMD model offers a tool for the successful analysis of the effects of drugs on cell markers of LMD proliferation and apoptosis.

### 4.3. Prospective Applications of the Zebrafish TNLMD Model

Future studies using the zebrafish TNLMD model could investigate TNBC cell metastasis in the leptomeninges, the metabolism of these cells in the leptomeningeal space, and the effects of novel anticancer drugs in combination with *in vivo* bioluminescent high-throughput fluorescent imaging. Studies using human-patient-derived samples have shown that metastasis in LMD often involves alteration of the expression of the cell adhesion protein E-cadherin, which leads to promotion of angiogenesis and invasion [35]. Fluorescent labeled MDA-MB-231 cells have been microinjected into *Tg* (*fli1*:*EGFP*) zebrafish embryos and used to show that Smad6 and Bmp6 expression regulates cancer cell invasion and is linked to E-cadherin expression [50]. Because the zebrafish TNLMD model provides the user with the ability to use fluorescent labeled TNBC cells in conjunction with protein marker stains (Figure 4 and Figure 5; Videos S2–S5), it should be readily adaptable for use with transgenic zebrafish that express fluorescent protein markers in the leptomeninges, to provide insight into the mechanisms governing fluorescent labeled TNBC cell metastasis in LMD in real time. Similarly, the model has great potential for studying the metabolic behavior of TNBC cells in the LMD-associated CSF compartments. Recent studies have shown that in oxygen-poor and lipid-depleted environments, cancer cells may utilize increased acetyl-CoA synthetase 2 (ACSS2) expression to maintain a competitive advantage [51], and that hypoxia-induced factor 1-alpha (HIF1-α) and 2-alpha (HIF2-α) expression [44] may modulate brain tumor response to oxygen-depleted conditions. Therefore, due to its ability to analyze fluorescent markers, the TNLMD model could be adjusted to study how ACSS2 and HIF expression is modulated in TNBC cells engineered to express fluorescent markers of these proteins. As the MDA-MB-231-Luc/RFP cells used in this project can convert luciferin substrate into a bioluminescent signal that can be analyzed to assess drug effects on vascularization in xenograft models [52], this capability suggests that cells expressing a luciferase reporter could be studied in the zebrafish TNLMD model in bioluminescent plate-reader-based assays that allow real-time monitoring of tumor cell progression and drug response. Additionally, as bioluminescent luciferase–luciferin assays have been used to monitor cardiac tissue development in transgenic zebrafish [53], future applications of the TNLMD model could potentially include engineering of relevant tissues, e.g., cells of the leptomeninges with Luc reporter genes, and the study of the interactions of these cells with labeled and genetically modified TNBC cells to better understand the metastatic and metabolic interactions of the cells of the leptomeninges with TNBC cells.

## 5. Conclusions

In summary, we demonstrated that the zebrafish xenograft TNLMD model allows for monitoring of MDA-MB-231 tumor area and migration in compartments associated with the metastatic transition of TNBC cells in human LMD. Further, we were able to use the model to analyze the effect of the breast cancer drug doxorubicin on TNBC cell area and to measure the effects of this drug on cancer cell proliferation and apoptosis using molecular markers. We believe that the combination of tractability, visual accessibility, and ability to analyze key aspects of leptomeningeal biology present in this model makes it a valuable new addition to the study of TNBC cancer metastasis, molecular behavior, and drug development in LMD.

## Data Availability

Not applicable.

## Supplementary Materials

The following supporting information can be downloaded at: https://www.mdpi.com/article/10.3390/cells12070995/s1, Video S1: Movie showing intraventricular injection of RFP-expressing MDA-MB-231 cells in 2 dpf zebrafish embryos; Video S2: Movie showing combined z-stack image layers for DMSO-treated samples with RFP-, Ki67-, and DAPI-labeled MDA-MB-231-Luc/RFP cells; Video S3: Movie showing combined z-stack image layers for doxorubicin-treated samples with RFP-, Ki67-, and DAPI-labeled MDA-MB-231-Luc/RFP cells; Video S4: Movie showing combined z-stack image layers for DMSO-treated samples with RFP-, caspase-3-, and DAPI-labeled MDA-MB-231-Luc/RFP cells; Video S5: Movie showing combined z-stack image layers for doxorubicin-treated samples with RFP-, caspase-3-, and DAPI-labeled MDA-MB-231-Luc/RFP cells.
